# Tuberculosis care models for children and adolescents: a scoping review

**DOI:** 10.2471/BLT.22.288447

**Published:** 2022-10-11

**Authors:** Courtney M Yuen, Daria Szkwarko, Melanie M Dubois, Shumail Shahbaz, Katharine A Yuengling, Michael E Urbanowski, Paul A Bain, Annemieke Brands, Tiziana Masini, Sabine Verkuijl, Kerri Viney, Yael Hirsch-Moverman, Hamidah Hussain

**Affiliations:** a Division of Global Health Equity, Brigham and Women’s Hospital, 75 Francis Street, Boston, MA 02115, United States of America (USA).; b Warren Alpert Medical School, Brown University, Providence, USA.; c Division of Infectious Diseases, Boston Children’s Hospital, Boston, USA.; d Indus Hospital and Health Network, Karachi, Pakistan.; e ICAP at Columbia University, New York, USA.; f T.H. Chan School of Medicine, University of Massachusetts Chan Medical School, Worcester, USA.; g Countway Library, Harvard Medical School, Boston, USA.; h Global Tuberculosis Programme, World Health Organization, Geneva, Switzerland.; i Interactive Research and Development Global, Singapore, Singapore.

## Abstract

**Objective:**

To map which tuberculosis care models are best suited for children and adolescents.

**Methods:**

We conducted a scoping review to assess the impact of decentralized, integrated and family-centred care on child and adolescent tuberculosis-related outcomes, describe approaches for these care models and identify key knowledge gaps. We searched seven literature databases on 5 February 2021 (updated 16 February 2022), searched the references of 18 published reviews and requested data from ongoing studies. We included studies from countries with a high tuberculosis burden that used a care model of interest and reported tuberculosis diagnostic, treatment or prevention outcomes for an age group < 20 years old.

**Findings:**

We identified 28 studies with a comparator group for the impact assessment and added 19 non-comparative studies to a qualitative analysis of care delivery approaches. Approaches included strengthening capacity in primary-level facilities, providing services in communities, screening for tuberculosis in other health services, co-locating tuberculosis and human immunodeficiency virus treatment, offering a choice of treatment location and providing social or economic support. Strengthening both decentralized diagnostic services and community linkages led to one-to-sevenfold increases in case detection across nine studies and improved prevention outcomes. We identified only five comparative studies on integrated or family-centred care, but 11 non-comparative studies reported successful treatment outcomes for at least 71% of children and adolescents.

**Conclusion:**

Strengthening decentralized services in facilities and communities can improve tuberculosis outcomes for children and adolescents. Further research is needed to identify optimal integrated and family-centred care approaches.

## Résumé

**Objectif:**

Caractériser les modèles de prise en charge de la tuberculose les mieux adaptés aux enfants et adolescents.

**Méthodes:**

Nous avons mené une analyse exploratoire afin d'évaluer l'impact d'une prise en charge décentralisée, intégrée et articulée autour du noyau familial sur les résultats observés chez les enfants et adolescents traités pour une tuberculose, mais aussi de décrire les approches employées pour ces modèles de soins et d'identifier les principales lacunes dans les connaissances à ce sujet. Nous avons effectué des recherches dans sept bases de données dédiées à la littérature le 5 février 2021 (mise à jour le 16 février 2022), examiné les références de 18 revues publiées et demandé des informations issues d'études en cours. Nous avons inclus des études provenant de pays fortement touchés par la tuberculose, qui utilisaient un modèle de prise en charge digne d'intérêt et faisaient mention de résultats de diagnostic, de traitement ou de prévention pour une catégorie d'âge < 20 ans.

**Résultats:**

Nous avons identifié 28 études avec un groupe de comparaison pour mesurer l'impact, et nous avons ajouté 19 études non comparatives à une analyse qualitative des approches de prestation de soins. Parmi ces approches figuraient le renforcement des capacités dans les établissements de niveau primaire, la fourniture de services au sein des communautés, le dépistage de la tuberculose dans d'autres services de santé, le regroupement des traitements contre la tuberculose et le virus de l'immunodéficience humaine, la possibilité pour le patient de choisir son lieu de traitement et enfin, la mise à disposition d'une aide sociale ou économique. Développer à la fois les services de diagnostic décentralisés et les réseaux communautaires a permis de multiplier par sept le dépistage des cas dans neuf études, et d'optimiser les résultats de prévention. Nous n'avons trouvé que cinq études comparatives consacrées aux soins intégrés ou articulés autour du noyau familial. En revanche, 11 études non comparatives ont indiqué que le traitement avait été couronné de succès chez au moins 71% des enfants et adolescents.

**Conclusion:**

Renforcer les services décentralisés dans les établissements et communautés peut contribuer à améliorer l'état des enfants et adolescents souffrant de tuberculose. D'autres recherches sont nécessaires pour dégager les meilleures approches intégrées et articulées autour du noyau familial.

## Resumen

**Objetivo:**

Establecer qué modelos de atención a la tuberculosis son los más adecuados para los niños y adolescentes.

**Métodos:**

Se realizó una revisión exploratoria para evaluar los impactos de la atención descentralizada, integrada y orientada a la familia en los desenlaces relacionados con la tuberculosis en niños y adolescentes, describir los enfoques de estos modelos de atención e identificar las principales deficiencias de conocimiento. Se realizaron búsquedas en siete bases de datos bibliográficas el 5 de febrero de 2021 (actualizadas el 16 de febrero de 2022), se buscaron las referencias de 18 revisiones publicadas y se solicitaron datos de estudios en curso. Se incluyeron estudios de países con una alta carga de tuberculosis que emplearan un modelo de atención de interés e informaran sobre los resultados de diagnóstico, tratamiento o prevención de la tuberculosis en un grupo de edad <20 años.

**Resultados:**

Se identificaron 28 estudios con un grupo comparativo para la evaluación del impacto y se añadieron 19 estudios no comparativos a un análisis cualitativo de los enfoques de prestación de atención. Los enfoques incluyeron el fortalecimiento de la capacidad en los centros de nivel primario, la prestación de servicios en las comunidades, la detección de la tuberculosis en otros servicios sanitarios, la ubicación conjunta del tratamiento de la tuberculosis y del virus de la inmunodeficiencia humana, la posibilidad de que el paciente elija el lugar de tratamiento y la prestación de apoyo social o económico. El refuerzo de los servicios de diagnóstico descentralizados y de los vínculos con la comunidad permitió multiplicar de una a siete veces la detección de casos en nueve estudios y mejorar los resultados de la prevención. Solo se identificaron cinco estudios comparativos sobre la atención integrada u orientada a la familia, pero 11 estudios no comparativos informaron de resultados de tratamiento exitosos en al menos el 71% de los niños y adolescentes.

**Conclusión:**

El refuerzo de los servicios descentralizados en los centros y las comunidades puede mejorar los desenlaces de la tuberculosis en niños y adolescentes. Es preciso seguir investigando para determinar los enfoques óptimos de atención integrada y orientada a la familia.

## ملخص

**الغرض:**

تحديد أفضل نماذج رعاية السل بالنسبة للأطفال والمراهقين.

**الطريقة:**

قمنا بإجراء مراجعة عن كثب لتقييم تأثير الرعاية اللامركزية المتكاملة التي تركز على الأسرة، على النتائج المتعلقة بالسل بالنسبة للأطفال والمراهقين، ووصف الاساليب الخاصة بنماذج الرعاية هذه، وتحديد الفجوات المعرفية الرئيسية. قمنا بالبحث في سبع قواعد بيانات للمنشورات في 5 فبراير/شباط 2021 (تم تحديثها في 16 فبراير/شباط 2022)، والبحث في مراجع لعدد 18 مراجعة منشورة وطلبنا البيانات من الدراسات الجارية. وكذلك قمنا بتضمين دراسات من الدول ذات العبء المرتفع من السل، والتي استخدمت نموذجًا مهمًا للرعاية، وأبلغت عن نتائج تشخيص السل أو علاجه أو الوقاية منه للفئة العمرية الأقل من 20 سنة.

**النتائج:**

قمنا بتحديد 28 دراسة مع مجموعة مقارنة لتقييم التأثير، وأضفنا 19 دراسة غير مقارنة إلى التحليل النوعي لأساليب تقديم الرعاية. تضمنت الأساليب تعزيز القدرات في مرافق المستوى الأولي، وتقديم الخدمات في المجتمعات، والكشف عن مرض السل مع الخدمات الصحية الأخرى، والاشتراك في اكتشاف علاج السل وفيروس نقص المناعة البشرية، وعرض اختيار المريض لمكان العلاج، وتقديم الدعم الاجتماعي أو الاقتصادي. أدى تعزيز كل من خدمات التشخيص اللامركزية والروابط المجتمعية إلى زيادات من واحد إلى سبعة أضعاف في اكتشاف الحالات عبر تسع دراسات، كما أدى لتحسين نتائج الوقاية. قمنا بتحديد خمس دراسات مقارنة فقط حول الرعاية المتكاملة أو التي تركز على الأسرة، لكن 11 دراسة غير مقارنة أفصحت عن نتائج علاجية ناجحة على الأقل لـ %71 من الأطفال والمراهقين.

**الاستنتاج:**

يمكن أن يؤدي تعزيز الخدمات اللامركزية في المرافق والمجتمعات إلى تحسين نتائج مرض السل بالنسبة للأطفال والمراهقين. هناك حاجة لمزيد من الأبحاث لتحديد أساليب الرعاية المثلى المتكاملة والتي تركز على الأسرة.

## 摘要

**目的:**

探讨哪种结核病护理模式最适合儿童和青少年。

**方法:**

我们开展了一项范围评审,评估分散式、综合和以家庭为中心的护理对儿童和青少年结核病相关护理效果的影响,介绍适用于此类护理模式的方法并确定关键的知识差距。我们于 2021 年 2 月 5 日从七个文献数据库中搜索了 18 份已出版评审的参考资料(于 2022 年 2 月 16 日进行了更新),并提取了目前正在开展的研究中的数据。我们纳入了存在较高结核病负担国家的研究,采用我们感兴趣的护理模式开展研究,并报告了 20 岁以下人群的结核病诊断、治疗或预防效果。

**结果:**

我们确定了 28 项采用对照组开展的影响评估研究和 19 项未采用对照组开展的研究,对护理方法进行了定性分析。方法包括加强初级护理机构的护理能力、面向社区提供服务、通过其他卫生服务开展结核病筛查、在同一地点协同开展结核病和艾滋病治疗、让患者选择治疗地点以及提供社会或经济支持。九项研究表明,通过加强分散式诊断服务与社区之间的联系,病例检测率提升了一至七倍,并且预防效果也显著提高。我们仅确定了五项综合或以家庭为中心护理方面的对比研究,但是 11 项非对比研究显示儿童和青少年的成功治疗结果至少提高 71%。

**结论:**

加强护理机构和社区内的分散式服务,可提高儿童和青少年的结核病护理效果。需开展进一步研究以确定最佳的综合和以家庭为中心的护理方法。

## Резюме

**Цель:**

Определить, какие модели лечения туберкулеза лучше всего подходят для детей и подростков.

**Методы:**

Для оценки влияния децентрализованного, интегрированного и ориентированного на семью ухода на результаты лечения туберкулеза у детей и подростков, описания подходов к этим моделям ухода и выявления основных пробелов в знаниях была подготовлена концептуальная проработка. 5 февраля 2021 года был проведен поиск в семи базах данных литературы (обновлено 16 февраля 2022 года), просмотрены ссылки на 18 опубликованных обзоров и запрошены данные текущих исследований. Были включены исследования из стран с высоким уровнем заболеваемости туберкулезом, в которых использовалась интересующая модель ухода и сообщалось о результатах диагностики, лечения или профилактики туберкулеза в возрастной группе младше 20 лет.

**Результаты:**

Для оценки воздействия было выделено 28 исследований с группой сравнения и добавлено 19 несравнительных исследований для качественного анализа подходов к оказанию медицинской помощи. В качестве подходов использовались: укрепление потенциала учреждений первичного звена, предоставление услуг в общинах, скрининг на наличие туберкулеза в других медицинских учреждениях, совместное лечение туберкулеза и вируса иммунодефицита человека, предоставление пациенту возможности выбора места лечения и оказание социальной или экономической поддержки. Развитие децентрализованных диагностических служб и укрепление связей с местными общинами привели к увеличению выявления случаев заболевания от одного до семи раз в девяти исследованиях и улучшению результатов профилактики. Было выявлено только пять сравнительных исследований по интегрированному или ориентированному на семью уходу, однако в 11 несравнительных исследованиях сообщалось об успешных результатах лечения не менее 71% детей и подростков.

**Вывод:**

Развитие децентрализованных услуг в учреждениях и общинах может способствовать улучшению результатов лечения туберкулеза у детей и подростков. Для определения оптимальных подходов к интегрированному и ориентированному на семью уходу необходимо проведение дальнейших исследований.

## Introduction

Of the roughly 10 million people who develop active tuberculosis annually, around one in every six is a child or adolescent aged 0–19 years old.^1^^,^^2^ In 2020, less than half of the children with tuberculosis were diagnosed and treated, and only an estimated 36% of young child contacts eligible for tuberculosis preventive treatment received it.^1^

When considering how to improve the detection, treatment and prevention of tuberculosis in children and adolescents, policy-makers must recognize that children and adolescents have different health care needs from adults. Tuberculosis diagnosis in children is challenging given the overlap in symptoms with common childhood illnesses, and the higher likelihood of paucibacillary and disseminated disease makes bacteriologic testing difficult.^3^ Children and adolescents often access the health system differently from adults, as they may attend paediatric or youth clinics and their access may be dependent on a guardian.

Care models that remove barriers to accessing services or completing treatment can help ensure that children and adolescents are diagnosed promptly, treated effectively and receive appropriate preventive care. Three broad strategies that seek to reduce barriers are decentralization of care, integration of care and family-centred care.^4^ Decentralization refers to provision of services at points in the health system where patients first seek care. These points of first contact are often primary-level or community health centres, outpatient clinics or private general practitioners rather than specialized tuberculosis clinics or hospitals. Integration refers to coordinating care for multiple health conditions. Family-centred care is responsive to the needs of the family affected by tuberculosis. Important components of family-centred care are offering families a choice in what treatment they receive or how it is delivered, as well as addressing their social, psychological and economic needs.

Most of the literature evaluating decentralized, integrated or family-centred care models for tuberculosis has not specifically addressed children or adolescents. A systematic review of adherence interventions for children and adolescents showed that community-based and family-centred interventions promote successful tuberculosis disease treatment;^5^ however, this review did not encompass diagnostic or prevention outcomes. Other systematic reviews have assessed the impact of community-based case-finding,^6^^,^^7^ decentralized care for multidrug-resistant tuberculosis,^8^ community-based treatment support,^9^ integration of tuberculosis and human immunodeficiency virus (HIV) services^10^^,^^11^ and socioeconomic and psychosocial support.^12^^–^^14^ However, these reviews have not sought to disaggregate child or adolescent outcomes from adult outcomes and many of the included studies focus on adults. Because children and adolescents have unique needs based on clinical and life stage considerations, it is unclear whether the impact observed for adults translates to children and adolescents.

To address this knowledge gap, we conducted a scoping review to assess the evidence for the impact of decentralized, integrated and family-centred care on child and adolescent tuberculosis outcomes in countries with high tuberculosis burdens. Our objectives were to (i) quantitatively assess the impact of these care models on child and adolescent tuberculosis diagnosis, treatment and prevention outcomes; (ii) describe the varied approaches to implementing these care models; and (iii) identify key gaps in knowledge around the impact of these care models.

## Methods

Our objectives were to map out the available evidence around a diverse set of care models and help define how these care models are being implemented in tuberculosis services for children and adolescents. We chose a scoping review because this method is more appropriate than a systematic review for exploring the definitions of decentralized, integrated and family-centred care as they apply to tuberculosis care delivery rather than assessing the evidence around a specific set of approaches defined a priori.^15^

WHO staff members defined the research question for the review and commissioned an independent group of experts (CMY, HH, YHM, DS) to conduct it. This group of experts submitted a study protocol to WHO for approval before conducting the review.

### Search strategy

To develop our search strategy, we first defined key features of decentralized, integrated and family-centred care in consultation with four WHO staff members and three stakeholders with experience working in middle-income country tuberculosis programmes. We developed search terms based on the results of these discussions and by consulting published systematic reviews. We searched PubMed®, Embase®, Web of Science™, WHO regional databases of the Global Index Medicus, Global Health and the Cochrane Central Register of Controlled Trials on 5 February 2021. We reviewed a sample of the first 400 abstracts and 45 articles from the database search to better define the care models, consulting stakeholders to resolve ambiguity. Based on our refined definitions, we supplemented the database search by searching the reference lists of systematic and non-systematic reviews and requesting unpublished data from ongoing studies. The development of the search strategy and search terms are available in our data repository.^16^ We updated the PubMed® search on 16 February 2022, as all the included studies identified in the original database search were found in PubMed®.

### Study selection

We defined seven outcomes of interest related to diagnosis (case notifications in a geographical area, diagnoses in a cohort and delay in tuberculosis diagnosis), treatment (successful treatment for tuberculosis disease) and prevention (tuberculosis preventive treatment initiation, delay in such initiation and tuberculosis preventive treatment completion among contacts). We did not include tuberculosis preventive treatment among children or adolescents living with HIV. We considered individuals aged 0–19 years, encompassing children (0–9 years) and adolescents (10–19 years). To identify feasible approaches for programmes managing large numbers of people with tuberculosis disease or exposure, we limited the review to 74 countries that either had an estimated tuberculosis incidence of ≥ 100 cases per 100 000 population in the 2020 WHO Global Tuberculosis Report (64 countries)^17^ or appeared on WHO’s list of tuberculosis priority countries for 2016–2020 (48 countries).^18^

Two authors reviewed abstracts and full-text articles, and any disagreements were arbitrated by a third reviewer. In the abstract review, we included those that reported any outcome of interest and excluded those restricted to populations aged 18 years or older (a conventional definition of adults) since these papers would be unlikely to disaggregate data for just the adolescents 18–19 years old. In the full-text review, for the quantitative assessment, we included comparative studies that reported outcomes of interest for a group that received decentralized, integrated or family-centred care and a group that did not. We also identified non-comparative studies for the qualitative assessment. We included articles in any language. Inclusion and exclusion criteria are available in the data repository.^16^

### Outcome data extraction

We extracted data on study design and setting, care model features and outcomes for available age groups within the 0–19 year range. We extracted numbers of events and people in control and intervention groups, and case notifications for intervention and pre-intervention periods in intervention and control areas. For comparative studies we performed quality assessments with the Cochrane Risk of Bias 2 tool for randomized studies or an adapted Newcastle–Ottawa scale for non-randomized studies (available in the data repository).^16^

### Qualitative analysis 

We used a qualitative analysis approach to group interventions into general approaches for evidence synthesis. We assigned codes to intervention components, grouped codes into themes corresponding to general approaches, then grouped these approaches under the parent themes of decentralized, integrated or family-centred care models. For studies reflecting multiple care models, we categorized the study according to the predominant care model described by the authors. We included non-comparative studies in the qualitative analysis for care models where there were fewer than five comparative studies identified. While these studies would not address the impact of the care model, they could contribute to the second objective of describing care delivery approaches.

### Calculation of effect estimates

We calculated risk ratios (RR) or incidence rate ratios (IRR) and corresponding 95% confidence intervals (CIs) for the child and adolescent age group. For cohort studies, we calculated RR based on count data. For studies where the outcome was case notifications, we estimated annual IRR based on case notifications during the intervention and pre-intervention periods, assuming the size of the underlying population to remain constant. Where possible, we calculated IRRs adjusted for changes in case notification rate over time in a control area (i.e. the ratio of IRRs between the intervention and control area). To estimate CIs for unadjusted IRRs, we used a large-sample normal approximation. We report exact CIs for estimates based on small numbers of events. Statistical analysis was performed in SAS version 9.4 (SAS Institute Inc., Cary, United States of America).

## Results

### Study identification

**Fig. 1:**
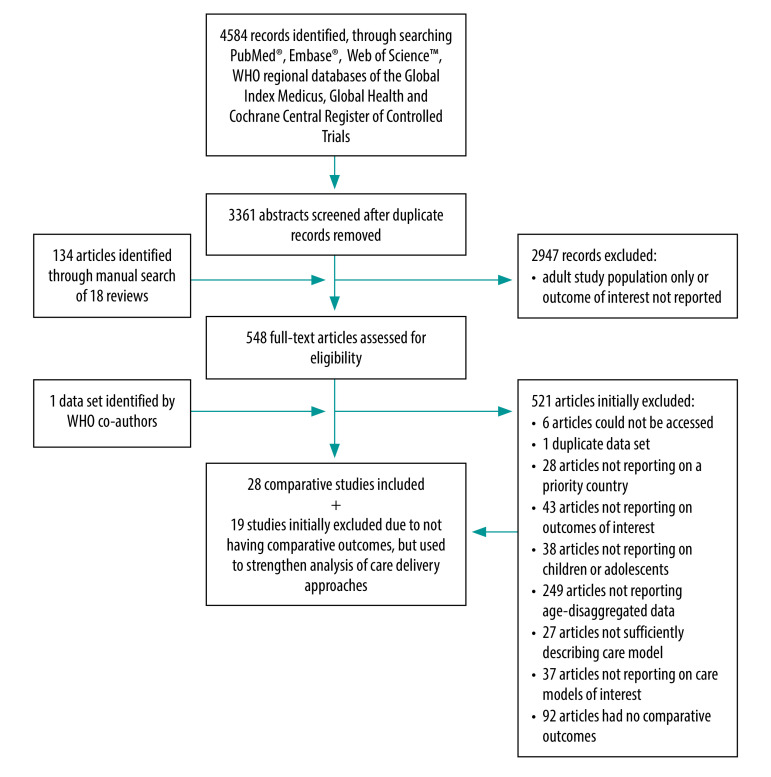
Flowchart of the selection of articles included in study on tuberculosis care models for children and adolescents

Note: We performed the literature search 5 February 2021 and updated the search 16 February 2022. We identified three out of the 47 included studies (two comparative and one non-comparative) in the search update.

We reviewed 3361 abstracts from database searches and an additional 134 studies referenced by 18 reviews (Fig. 1). We identified 27 published comparative studies^19^^–^^45^ and one unpublished data set.^46^ Many studies described multifaceted interventions and interventions were heterogeneous (Table 1; available at: https://www.who.int/publications/journals/bulletin/). The primary intervention was decentralization for 23 studies,^19^^–^^39^^,^^44^^,^^46^ integration for three studies^40^^,^^41^^,^^45^ and family-centred care for two studies.^42^^,^^43^ Because of the small number of studies of integrated and family-centred care models, we incorporated into the evidence synthesis 19 additional studies without comparative outcomes (six studies reporting on integrated models^47^^–^^52^ and 13 studies reporting family-centred models;^53^^–^^65^ Table 2; available at: https://www.who.int/publications/journals/bulletin/).

**Table 1:** Characteristics of comparative studies included in the scoping review on tuberculosis care models for children and adolescents

Study	Country; setting	Study design	Population	Intervention	Comparator	Comparative outcomes (age groups available for children or adolescents)
Bayona et al., 2003^28^	Peru; Lima city	Prospective cohort study with concurrent comparator	Household contacts (all ages) of patients treated for MDR tuberculosis in an individualized treatment programme (North Lima) or a standardized MDR tuberculosis treatment programme (Central Lima)	• Home visit for contact screening made when the index patient was enrolled • Follow-up home visits for contact screening performed by the DOT workers supporting the index patients throughout the index patient’s course of treatment; contacts with symptoms were referred for evaluation	• When index patient was enrolled, symptomatic household contacts were expected to go to health centre on their own • No home visits; symptomatic contacts were expected to go to health centre on their own	Tuberculosis cases diagnosed among contacts (0–14 years)
Datiko & Lindtjørn, 2009^37^	Ethiopia; Dale and Wonsho, rural districts of Sidama zone	Cluster-randomized trial (cluster: communities)	Patients (all ages) treated for smear-positive tuberculosis from 30 intervention communities and 21 control communities; in both arms, 10% of patients were children 0–14 years old	• Community DOT: tuberculosis patients received DOT from health extension workers at health posts, which are community-based health facilities and the most decentralized level of health system • Training (2-day) on identifying tuberculosis symptoms, collecting sputum, administering DOT and treatment support provided to health-care workers, laboratory technicians and health extension workers • Health extension workers gave tuberculosis-related health education	• Tuberculosis patients received DOT from health-care workers at health stations and health centres (more centralized facilities compared with health posts) • Training provided to health-care workers but not health extension workers (same content as in intervention arm) • Health extension workers gave tuberculosis-related health education	Patients with treatment success (0–14 years)
Davis et al., 2019^30^	Uganda; Kampala city	Cluster-randomized trial (cluster: household)	Household contacts (all ages) of patients newly diagnosed with tuberculosis at seven public primary care clinics	• Home visits for contact screening were made by CHWs • All contacts with any tuberculosis symptom, who were < 5 years old or who were living with HIV, had sputum collected in the home and were offered HIV testing in the home • Sputum test results and follow-up instructions were delivered to participants via an automated SMS system	• Home visits for contact screening were made by CHWs • All contacts with any tuberculosis symptom, who were < 5 years old or who were living with HIV, were referred to nearby health facilities for sputum testing, HIV testing and clinical evaluation	Tuberculosis cases diagnosed out of contacts (secondary trial outcome) (0–4, 5–14 years)
EGPAF, 2018^46^	Cameroon, Côte D’Ivoire, Democratic Republic of the Congo, India, Kenya, Lesotho, Malawi, Uganda, United Republic of Tanzania, Zimbabwe	Pre-post study without control area	Children and adolescents 0–14 years old with tuberculosis signs/symptoms attending study health facilities and contacts (0–14 years old for case-finding, 0–4 years old for preventive treatment)	• Training on paediatric tuberculosis and supportive supervision for health-care workers. Topics included sample collection, Xpert® MTB/RIF assay, management • Screening in primary care settings (waiting areas of outpatient department and paediatric wards) and screening in integrated settings (HIV, maternal–child health, nutrition clinics). Screening was performed by health-care workers or CHWs. Children with symptoms were referred for clinical evaluation • Home visits for contact screening performed by CHWs, who referred for evaluation or preventive treatment • Supplies for sample collection provided • Introduction of contact investigation and preventive treatment register, caregiver education materials for preventive treatment	• No explicit description of the pre-intervention care model is given, but pre-intervention situation is likely to have been variable across 10 study countries	People who initiated tuberculosis treatment at study facilities, contacts who initiated preventive treatment at study facilities (0–14 years for tuberculosis treatment, 0–4 years for preventive treatment)
Fatima et al., 2016^29^	Pakistan; Lahore, Rawalpindi, Faisalabad and Islamabad districts, which have high concentrations of slums	Pre-post study without control area	Household contacts (all ages) and people living within 50 m of patients with smear-positive tuberculosis (community contacts)	• Screening in homes was done by field workers who visited all residences within 50 m of a patient with smear-positive tuberculosis • Children with symptoms were referred to a health facility for evaluation, while adults with symptoms had sputum collected in the home	• Passive case-finding (no additional description)	Tuberculosis cases notified in study districts (0–14 years)
Hanrahan et al., 2019^27^	South Africa; Vhembe and Waterberg districts of Limpopo Province, largely rural	Cluster-randomized trial (cluster: health facilities)	Household contacts (all ages) of tuberculosis patients from study health facilities; health facilities (28 intervention, 28 control) comprised those providing essential services and those providing maternity, emergency and 48-hour inpatient care	• Home visits for contact screening were implemented in 14 facilities. Up to three home visits were made by the study team, with symptom screening and sputum collected in the home for those with symptoms • Incentive-based contact tracing was implemented in 14 facilities. Both index patients and contacts received monetary incentives if the contact went to the health facility • Passive case-finding with symptom-based screening was also done at health facility	• Passive case-finding with symptom-based screening for all people presenting to health facility	People who initiated tuberculosis treatment in study facilities (0–5, 6–20 years)
Islam et al., 2017^19^	Bangladesh; one urban slum area in Dhaka, two rural sites in Kalai and Bashail subdistricts	Pre-post study without control area	Children with tuberculosis signs or symptoms attending selected primary care facilities	• Training (twice per year) on child tuberculosis management was provided for graduate doctors, field workers, laboratory technicians, radiologists, medical assistants, village doctors and drug sellers • Community awareness of childhood tuberculosis was promoted through community health education sessions with mothers of young children, teachers, students and religious and community leaders • Procurement support via quarterly meetings with local managers	• Primary care health workforce had no training on childhood tuberculosis management • Primary care facilities experienced shortages of tuberculin and radiology supplies	Tuberculosis cases at study facilities; unclear if reported outcome is diagnosis or treatment initiation (no definition of children is stated)
Jeena & Naidoo, 2016^34^	South Africa; peri-urban community including low-cost state housing and informal settlements	Retrospective cohort study with historic comparator	Tuberculosis patients 0–15 years old who received doorstep or clinic-based tuberculosis care	• Home visits to patients made by community caregivers or nurses 2–3 times per week. At visits, they recorded basic clinical history, evaluated adherence and addressed adherence issues, offered tuberculosis education and dispensed medications	• Patients received care from primary care clinics. Patients visited clinics once a month to collect medications	Standard tuberculosis disease treatment outcomes (< 1, 1–6, 7–12, 13–15 years)
Joshi et al., 2015^20^	Nepal; seven intervention districts having high poverty, higher population density and lower case notification rates compared with the national average; seven control districts had no active case-finding activities being implemented	Pre-post study with control area	Household contacts 0–14 years old and children and adolescents 0–14 years old with tuberculosis signs or symptoms	• Home visits for contact screening (0–14 years) performed by volunteers, with referral to health facilities or sputum collected in the home • Screening in communities via mobile camps equipped with doctors, microscopy and tuberculin skin test • Screening in homes for families with a member living with HIV performed by volunteers • Screening in schools performed by volunteers • Screening in maternal–child health clinics performed by maternal–child health providers; children with tuberculosis symptoms referred to tuberculosis centre for diagnosis • Private sector practitioners were reimbursed for tuberculosis diagnoses	• No active case-finding activities were being implemented	Tuberculosis cases notified in study districts (0–4, 5–14 years)
Ketema et al., 2020^41^	Ethiopia; Addis Ababa city	Stepped-wedge trial (cluster: health facilities)	Children 0–4 years old with tuberculosis signs or symptoms attending integrated maternal, neonatal and child illnesses clinics in 30 health facilities	• Training (3-day) on childhood tuberculosis for integrated maternal, neonatal and child illnesses health-care workers • Supplies: reference materials, nasogastric aspiration supplies and new integrated maternal, neonatal and child illnesses registers with a tuberculosis screening column were provided • This trial also included a component of training health-care workers in the tuberculosis clinic on childhood tuberculosis, but this did not represent a care model of interest, so only the integrated maternal, neonatal and child illnesses outcomes were considered	• No description of pre-intervention standard practice in integrated maternal, neonatal and child illnesses clinics with respect to tuberculosis screening	Tuberculosis cases diagnosed among children attending integrated maternal, neonatal and child illnesses clinics (0–4 years)
Khan et al., 2012^21^	Pakistan; Korangi and Bin Qasim areas of Karachi comprised the intervention area; Landi and Shah Faisal areas of Karachi comprised the control area. Both are lower-income urban areas	Pre-post study with control area	People (all ages) with tuberculosis signs or symptoms attending Indus Hospital (a private free hospital that is also a national tuberculosis programme reporting centre) or 50 private family clinics (primary care facilities) in the intervention area	• Screening in primary care settings performed by incentivized screeners (community members hired for the intervention) for all attendees of 50 private family clinics and the Indus Hospital outpatient department • Anyone with prolonged cough, prior tuberculosis history or a family member with tuberculosis was evaluated for tuberculosis. Children were referred to Indus Hospital specialists, while adults had spot sputum collected at the screening point • Community awareness promoted through advertisements, posters, flyers, banners, etc. encouraging people with ≥ 2 weeks of cough to seek care at family clinics or Indus Hospital	• No explicit description of the pre-intervention care model is given	Tuberculosis cases notified by Indus Hospital, one of the five national tuberculosis programmes reporting centres in the intervention area (0–14 years)
Maha et al., 2019^22^	Papua New Guinea; East New Britain Province	Pre-post study without control area	People (all ages) with tuberculosis signs or symptoms attending the provincial hospital (centralized) and two decentralized health facilities	• Training courses on tuberculosis management, HIV testing and integrated management of adult illness was conducted for health-care workers • Hospital specialists provided weekly medical consultation and patient review for decentralized facilities • Xpert® MTB/RIF testing was made available for all presumptive tuberculosis cases • Community awareness campaign to encourage recognition of tuberculosis symptoms	• Passive, facility-based case-finding • The Xpert® MTB/RIF assay was available at the hospital, while the two decentralized facilities had only smear microscopy	People who initiated tuberculosis treatment in study facilities (0–14 years)
Malik et al., 2018^23^	Pakistan; Jamshoro, a rural district, was the intervention area; Hyderabad, a neighbouring district with similar demographics, was the control area	Pre-post study with control area	People (all ages) with tuberculosis signs or symptoms attending outpatient departments of three general hospitals and one chest clinic	• Screening in primary care settings performed by CHWs in general, paediatric and chest outpatient departments. People with symptoms or contact with tuberculosis patient were referred for evaluation • Transport enablers were given to households of tuberculosis patients for bringing contacts to the health facility for screening • Training of medical officers in childhood tuberculosis diagnosis and management • Community awareness: advertisements on television and signboards	• No explicit description of the pre-intervention care model is given	Tuberculosis cases notified in study districts (0–14 years)
Mathew et al., 2005^35^	India; Palamu district in Jharkhand state, a poor district with a large tribal population	Retrospective cohort study with concurrent comparator	Patients (all ages) treated at Nav Jivan Hospital	• Patients went to hospital once every 2 months • Community DOT: a member of the community, not a family member, was recruited to keep medications and directly observe treatment • Drugs were free • Patients paid a 300-rupee deposit which would be refunded after completion of treatment	• Patients went to hospital monthly • A family member was expected to supervise treatment • Patients paid for drugs • Patients paid a 300-rupee deposit which would be refunded after completion of treatment	Standard tuberculosis disease treatment outcomes (0–14 years)
Miyano et al., 2013^40^	Zambia; Mumbwa district, rural	Pre-post study with control facilities	Unclear; outcome is tuberculosis patients (all ages) who enrolled in treatment, but how these patients were diagnosed and how they entered care is not discussed	• Tuberculosis diagnostic and treatment services offered at decentralized rural health centres • Co-location of ART: HIV diagnosis and ART available at rural health centres	• Tuberculosis diagnostic and treatment services offered at decentralized rural health centres (same as intervention) • HIV diagnosis available at rural health centres, but ART only available at hospitals	People who initiated tuberculosis treatment in study facilities (0–14 years)
Moyo et al., 2012^31^	South Africa; Cape Winelands District	Randomized trial	Healthy infants vaccinated with bacille Calmette–Guérin within 72 hours of birth were enrolled within 2 weeks of birth	• Home visits for screening were made every 3 months by the study team. Children with a close tuberculosis contact or symptoms were evaluated at a study clinic • Tuberculosis registers of study area clinics were monitored. If an adult tuberculosis patient was identified as a contact of a study participant or a participant was diagnosed with tuberculosis, the participant was evaluated at a study clinic • Hospital admission and X-ray department records were reviewed to identify potential tuberculosis-related conditions. Participants with these conditions were evaluated at a study clinic	• No home visits • Monitoring of tuberculosis registers, hospital admission records and X-ray department records was performed as for the intervention group	Tuberculosis cases diagnosed per person-years of follow-up (0–6, 6–12, 12–18, 18–26 months)
Oshi et al., 2016^24^	Nigeria; Akwa Ibom, Rivers, Enugu, Ebonyi, Ogun and Lagos states; six control states chosen from the same geographical regions	Pre-post study with control area	Children/adolescents 0–14 years old with tuberculosis signs or symptoms attending 30 study health facilities and household contacts 0–14 years old of patients with smear-positive tuberculosis	• Training on screening, diagnosis and management of childhood tuberculosis for medical officers, paediatricians, nurses and general health workers • Training of medicine vendors on identification of presumptive child tuberculosis • Screening in primary care settings (general and child outpatient clinics) and screening in ART clinics was performed by nurses, general health workers and dedicated screeners. Children with symptoms were referred for evaluation • Home visits for contact screening were performed (but unclear by whom) • Community awareness of childhood tuberculosis was promoted via handbills and posters and education in primary schools • Supplies: tuberculin was procured and distributed	• No explicit description of the pre-intervention care model is given	Tuberculosis cases notified in study districts (0–14 years)
Oxlade et al., 2021^44^	Indonesia; Bandung^a^	Cluster-randomized trial (cluster: health facilities)	Household contacts of tuberculosis patients at seven intervention and eight control primary health clinics	• Training on household contact identification, evaluation and latent tuberculosis infection diagnosis given once at the beginning and reinforced twice per month • Educational flip charts and contact management register introduced into health facilities • Periodic care cascade performance reviews • Integrated database reminder system • Toys given to children attending visits	• No intervention	Contacts who initiated preventive treatment (0–4 years)
Reddy et al., 2015^32^	India; Kolar and Bidar districts of Karnataka state	Pre-post study with control area	People (all ages) with tuberculosis signs or symptoms who belong to lower caste or tribal groups	• Screening in homes and delivery of tuberculosis education was performed by trained community volunteers who visited homes of people belonging to vulnerable populations • People with symptoms were either referred to health facilities or had sputum collected in the home	• Passive case-finding (no additional description)	Smear-positive tuberculosis cases notified in study districts (0–14 years)
Rocha et al., 2011^43^	Peru; eight contiguous shanty towns in northern Lima	Pre-post study without control area	Tuberculosis patients (all ages) and their household contacts (all ages); preventive treatment outcomes assessed for contacts 0–19 years old, who are eligible for preventive treatment in Peru	• Psychosocial support activities included home visits, community mobilization workshops for groups of tuberculosis-affected households and psychological counselling • Food support • Economic support: cash transfers, microcredit loans, vocational training and microenterprise activities • All interventions were offered to all households, but patients and household members could choose whether to accept individual components	• No explicit description of the pre-intervention care model is given	Contacts who initiated preventive treatment among all contacts, contacts who completed preventive treatment (0–19 years)
Sachdeva et al., 2015^33^	India; 18 subdistricts in diverse geographical and demographic settings	Pre-post study without control area	People (all ages) with tuberculosis signs or symptoms attending all 99 microscopy centres and their associated primary care facilities (3–5 per microscopy centre) in study subdistricts	• People identified with tuberculosis symptoms in primary care facilities were referred to microscopy centres • Two sputum samples were collected and tested by Xpert® MTB/RIF assay at that microscopy centre or another one in the same subdistrict (one Xpert® machine per subdistrict)	• People identified with tuberculosis symptoms in primary care facilities were referred to microscopy centres • Two sputum samples collected for smear microscopy • Unclear whether there was centralized Xpert® MTB/RIF assay capacity, but study is described as a decentralization of Xpert® MTB/RIF testing	Pulmonary tuberculosis cases diagnosed among people tested for tuberculosis in study subdistricts (0–14 years)
Szkwarko et al., 2021^45^	Kenya; Bungoma county	Pre-post study without control area	Children 0–15 years old attending county hospital	• Screening in hospital paediatric outpatient clinics (nutrition, maternal–child health, acute care) was conducted in waiting rooms by trained CHWs equipped with a mobile phone screening app • Caregivers of children who screened positive were given a card that alerted the health-care worker to consider presumptive tuberculosis	• No screeners in waiting rooms of paediatric outpatient clinics	People registered for tuberculosis treatment at hospital tuberculosis clinic (0–15 years)
Talukder et al., 2012^25^	Bangladesh; 10 randomly selected districts where Damien Foundation was supporting microscopy centres	Cluster-randomized trial (cluster: microscopy centres)	Children and adolescents 0–14 years old attending 18 intervention microscopy and 18 control centres, including contacts and people with tuberculosis signs or symptoms	• Training (2-day) in child tuberculosis screening and diagnosis for tuberculosis and Leprosy Control Assistants, their supervisors, government health centre doctors, field coordinators and field workers • Supplies: microscopy centres were given flip charts for health education, scales and tuberculin • Community awareness activities, including distributing posters and pamphlets and disseminating messages about childhood tuberculosis at various types of community meetings • Introduction of a flowchart for screening and referral of children based on Keith Edwards Child Tuberculosis Score	• Training (2-day) in child tuberculosis screening and diagnosis for Leprosy Control Assistants and field coordinators, but not field workers or other cadres • The absence of the other intervention components (supplies, community awareness activities, flowchart for screening and referral) is assumed but not stated	People diagnosed with tuberculosis at study microscopy centres (0–14 years)
Tripathy et al., 2013^36^	India; Bangalore city	Retrospective cohort study with concurrent comparator	Patients (all ages) with new, smear-positive tuberculosis treated in public health services	• Community DOT: patient received treatment from a community DOT provider, defined as a person who volunteered to administer DOT and who belonged to the community where the patient resided, but was not a government health worker or a member of the family	• Patient received treatment from an institution DOT provider, defined as a government health worker at a health facility	Patients with treatment success (0–14 years)
Wingfield et al., 2017^42^	Peru; Callao shanty towns	Cluster-randomized trial (cluster: households)	Household contacts 0–19 years old of patients newly initiating tuberculosis treatment	• Social support via household visits and community meetings to educate, empower and reduce stigma among patients and contacts • Economic support: conditional cash transfers to defray hidden costs of being on treatment. Cash transfers were given for index patient adherence to tuberculosis, contacts being screened, contacts adhering to preventive treatment and engagement with social support activities	• No intervention	Contacts who initiated preventive treatment among all contacts (0–19 years)
Yassin et al., 2013^38^	Ethiopia; Sidama zone	Pre-post study without control area	Household contacts 0–4 years old are the population for which the extracted outcome is assessed; the target population of the intervention was much broader	• Field supervisors screened household contacts of patients with smear-positive tuberculosis and initiated preventive treatment for asymptomatic children < 5 years old • This study also included many other components aimed at using health extension workers to conduct tuberculosis screening and monitor tuberculosis patients in the community, increase microscopy capacity and raise community awareness. However, these were unlikely to affect the preventive treatment outcome	• Contact tracing and preventive treatment were recommended by the national tuberculosis programme but were not being implemented	Contacts who initiated preventive treatment (0–4 years)
Zachariah et al., 2003^39^	Malawi; Thyolo district, rural	Pre-post study without control area	Child contacts 0–5 years old of newly diagnosed patients with smear-positive tuberculosis being treated at the main public hospital in the district	• Home visits for contact screening were performed. Children ≤ 5 years old were given referral slips for chest X-ray at the hospital. Preventive treatment could be initiated following evaluation • Sputum collection in the home was performed for contacts with cough > 3 weeks	• Index patients were informed that any household contact with cough should come to the hospital for evaluation and any household contact aged ≤ 5 years old should come to the hospital for evaluation and preventive treatment initiation	Contacts who initiated preventive treatment among all contacts (0–5 years)
Zawedde-Muyanja et al., 2018^26^	Uganda; Kabarole (rural) and Wakiso (urban) districts	Pre-post study without control area	Children/adolescents 0–14 years old attending the 57 level III/IV health centres (larger health centres with laboratories) in the intervention districts, including contacts and people with tuberculosis signs or symptoms	• Training delivered by health-care workers from district hospitals (level V) to health-care workers from level III/IV health centres on child tuberculosis diagnosis, treatment and prevention. They also provided on-site mentorship and supportive supervision monthly initially and then quarterly • Training for health-care workers at level IV centres in sputum induction and gastric lavage; access to e-learning child tuberculosis course was given • Training (2-day) for CHWs on symptom screening and contact management • Home visits for contact screening and treatment support made by CHWs. Contacts referred for evaluation and preventive treatment • Procurement support given and microscopes repaired to improve clinic and laboratory functionality	• No explicit description of the pre-intervention care model is given, but 96% of childhood tuberculosis cases in the pre-intervention period were diagnosed by referral hospitals as opposed to health centres. The intervention is framed as decentralization of capacity for childhood tuberculosis management, so we assume that diagnostic and treatment capacity for childhood tuberculosis was centralized at the hospitals before the intervention	Tuberculosis cases notified in study districts, tuberculosis disease treatment outcomes (0–4, 0–14 years)

ART: antiretroviral therapy; CHW: community health worker; DOT: direct observed therapy; EGPAF: Elizabeth Glaser Pediatric AIDS Foundation; HIV: human immunodeficiency virus; SMS: short message service.

^a^ Data from Indonesia included in this review. Other sites were not countries of interest (Benin and Canada) or their preventive treatment results did not separate child contacts and contacts of all ages with HIV (Benin, Ghana and Viet Nam).

Note: Standard tuberculosis disease treatment outcomes are treatment success (completion or cure), treatment failure, loss to follow-up and death.

**Table 2:** Characteristics of non-comparative studies of integrated or family-centred care included in the scoping review on tuberculosis care models for children and adolescents

Study	Country; setting	Study design	Population	Intervention	Outcome (age groups available for children or adolescents)
Demissie et al., 2003^55^	Ethiopia; Este and Adet districts in Amhara state	Retrospective cohort study	Tuberculosis patients living in rural villages	• Social support via establishment of tuberculosis clubs of patients living in the same area	Standard tuberculosis disease treatment outcomes (0–14 years)
Dick et al., 1996^59^	South Africa; Cape Flats suburb, Western Cape	Retrospective cohort study	Tuberculosis patients	• Choice of treatment supporter to supervise DOT. Options included clinic staff and community volunteers • Choice of treatment locations included work, school or a specialized childcare centre	Patients who took ≥ 75% of doses in 6 months (0–14 years)
Htet et al., 2018^53^	Myanmar; Mandalay city	Prospective cohort study	Household contacts of tuberculosis patients receiving treatment for at least 3 months	• Transportation was provided to contacts to take them to and from health facility • Chest radiography was facilitated for all contacts	Tuberculosis cases diagnosed among contacts (0–14 years)
Kay et al., 2022^65^	Eswatini; Hhohho	Prospective cohort study	Household members of pulmonary tuberculosis patients whose households included a child younger than 5 years	• Choice of location for initial contact evaluation (facility or at home visit) • Asymptomatic child contacts < 5 years old offered 3-month isoniazid rifampicin preventive treatment regimen • Choice of location for preventive treatment management (facility or home-based)	Contacts who completed preventive treatment (0–4 years)
Imsanguan et al., 2020^54^	Thailand; Chiang Rai province	Prospective cohort study	Household and non-household contacts of tuberculosis patients	• Transport enablers provided to contacts for them to travel to a health facility for evaluation • Vouchers for free chest radiography provided to contacts (normally chest radiographs are not free)	Tuberculosis cases diagnosed among contacts (0–4, 5–18 years)
Mandalakas et al., 2020^50^	Botswana, Eswatini, Lesotho, Malawi, Uganda, United Republic of Tanzania; facilities affiliated with Baylor International Pediatric AIDS Initiative	Retrospective cohort study	Children and adolescents living with HIV who were receiving tuberculosis treatment	• Children received tuberculosis and HIV treatment at HIV care centres	Standard tuberculosis disease treatment outcomes (0–19 years)
Mehra et al., 2016^47^	India; Delhi city	Retrospective cohort study	Clients diagnosed with HIV at an HIV counselling and testing centre	• Screening of clients at HIV testing centre with referral of people with presumptive tuberculosis to tuberculosis clinic for evaluation	Tuberculosis cases diagnosed among people screened (0–14 years)
Mesic et al., 2020^62^	Afghanistan; Kandahar province	Retrospective cohort study	Patients with rifampicin-resistant tuberculosis	• Transport enablers for patients, caretakers and contacts • Accommodation provided for patients from outside Kandahar city • Counselling by trained counsellors • Social support via peer groups • Patient preferences between individualized and short regimens were considered	Patients with treatment success (0–14 years)
Oyieng’o et al., 2012^56^	Kenya; Western and North Rift provinces	Retrospective cohort study	Patients with MDR tuberculosis	• Home-based treatment support provided by a nurse where available • Transport enablers for patients • Food supplementation and access to other social services provided	Patients with treatment success (15 years; only one non-adult patient)
Patel et al., 2013^51^	Democratic Republic of the Congo; Kinshasa city	Prospective cohort study	Children and adolescents living with HIV who were receiving tuberculosis treatment	• Tuberculosis and HIV treatment provided at primary health centres during the same clinic visits	Standard tuberculosis disease treatment outcomes (3–18 years)
Qader et al., 2019^48^	Afghanistan; Kabul, Jalalabad, Kandahar, Herat and Mazar-e-Sharif provinces	Prospective cohort study	Patients being treated for mental health conditions at one private and five public mental health centres	• Symptom screening of patients registered in mental health centres conducted by nurses working in those centres • Home-based screening or telephone-based screening for some patients	Tuberculosis cases diagnosed among people screened (0–15 years)
Reif et al., 2018^52^	Haiti; Port-au-Prince city	Retrospective cohort study	Adolescents with microbiologically confirmed tuberculosis receiving care at an integrated tuberculosis and HIV clinic, most of whom were HIV-negative	• Tuberculosis and HIV treatment provided at the same integrated adolescent care clinic • Transport enablers • Social support via HIV peer educators • System of patient tracking established to contact patients who missed clinic appointments by phone or home visit	Standard tuberculosis disease treatment outcomes (10–15, 16–19 years)
Satti et al., 2012^57^	Lesotho, national cohort	Retrospective cohort study	Children and adolescents with MDR tuberculosis	• Community-based treatment support by trained and paid CHWs • Both tuberculosis and HIV treatment delivered by the same CHWs for patients with both tuberculosis and HIV • Transport enablers • Nutritional supplements provided • Economic support through helping families start income-generating activities • Counselling and psychosocial support • Assistance returning to school	Standard tuberculosis disease treatment outcomes (2–15 years)
Soomro et al., 2012^60^	Pakistan, Rawalpindi district	Retrospective cohort study	Patients with smear-positive tuberculosis registered in the public sector	• Choice of treatment supporter: options were health facility staff, CHWs, community volunteers, lady health workers and family members	Patients with treatment success (0–14 years)
Szkwarko et al., 2016^49^	Kenya, Eldoret city	Prospective cohort study	Street-connected young people	• Screening and sputum collection in congregate settings where street-connected young people sleep • Screening and sputum collection in centres providing services for street-connected young people by a symptom screener from the tuberculosis programme working with the centre’s medical outreach worker • Tuberculosis education provided at places where street-connected young people stay	Tuberculosis cases diagnosed among people screened (10–18 years)
Triasih et al., 2016^64^	Indonesia; Yogyakarta city	Prospective cohort study	Child contacts of tuberculosis patients	• Choice of location for preventive treatment: options were a primary health centre, lung clinic or hospital	Contacts who initiated preventive treatment among eligible contacts, contacts who completed preventive treatment (0–4 years)
van den Boogaard et al., 2009^61^	United Republic of Tanzania; Kilimanjaro region	Retrospective cohort study	Tuberculosis patients	• Choice of location for DOT: options were facility-based or community-based DOT	Patients with treatment success (0–14 years)
Wai et al., 2017^58^	Myanmar; 33 townships	Retrospective cohort study	Patients with MDR tuberculosis	• Home-based treatment support by a trained and paid community volunteer, who helps patient access treatment and coordinates specimen transport • Economic support through monthly cash transfer to offset expenses of lodging when visiting tuberculosis care centres and ancillary drugs • Food support • Counselling for patient, family and neighbours • Community awareness promoted by providing health education to patient, family and neighbours	Patients with treatment success (0–14 years)
Yuen et al., 2019^63^	Peru; Carabayllo district of Lima city	Prospective cohort study	Contacts of tuberculosis patients	• Home visits to encourage household contacts to complete evaluation • Home-based preventive treatment support with adherence counselling by a CHW and monthly visits by a nurse technician for adverse event monitoring • Transport enablers • Assistance coordinating monthly appointments	Contacts who initiated preventive treatment among all contacts, contacts who completed preventive treatment (0–19 years)

CHW: community health worker; DOT: direct observed therapy; HIV: human immunodeficiency virus; MDR: multidrug-resistant.

Note: Standard tuberculosis disease treatment outcomes are treatment success (completion or cure), treatment failure, loss to follow-up and death.

### Evidence synthesis

#### Decentralized care interventions

**Fig. 2:**
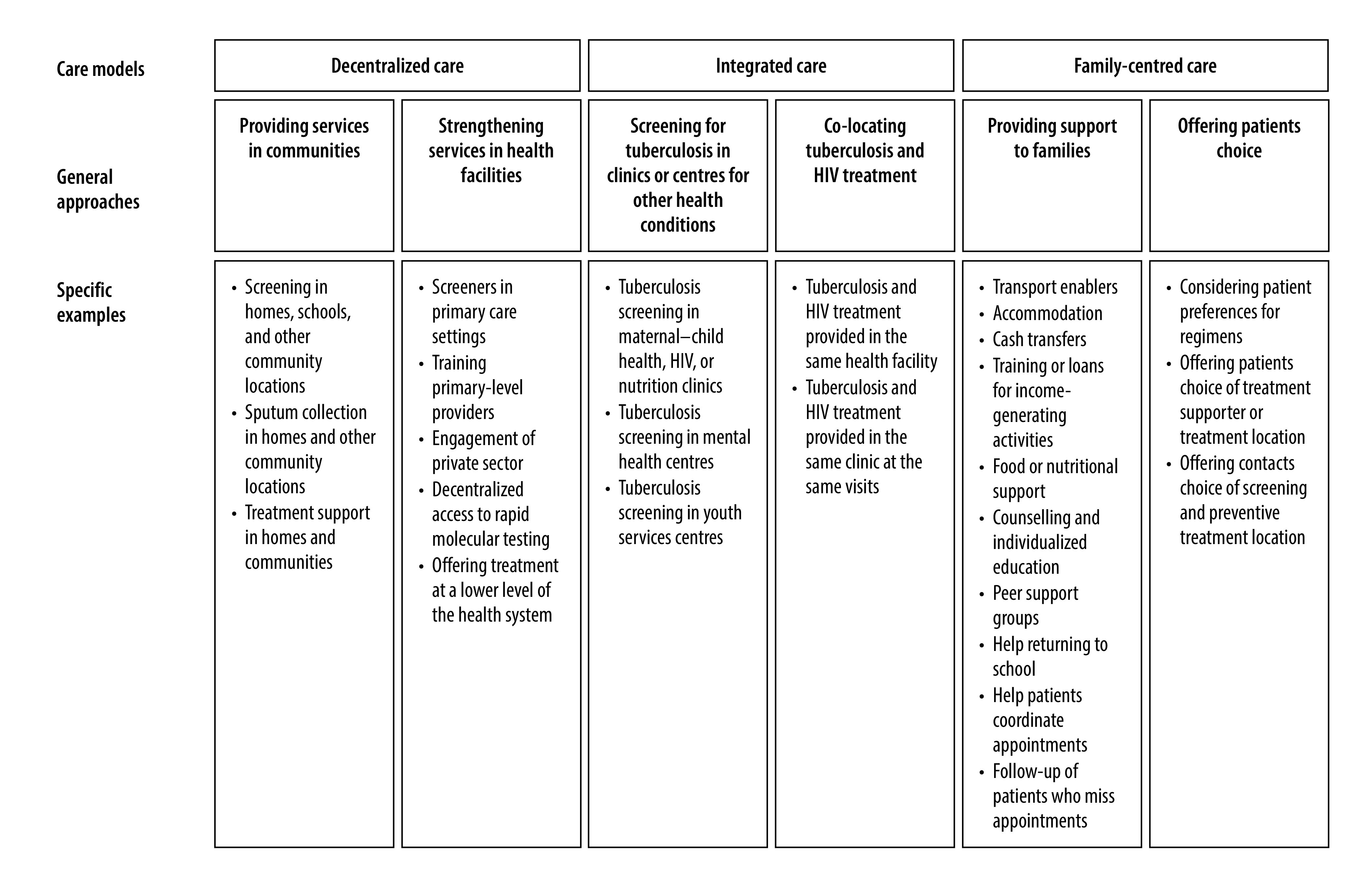
Tuberculosis care delivery approaches for children and adolescents

Of the 23 studies assessing the impact of a decentralized care model (Table 3),^19^^–^^39^^,^^44^^,^^46^ our thematic analysis identified two major intervention approaches: strengthening services within health facilities and providing services in communities (Fig. 2). Facility-based approaches included training primary-level providers in diagnosing and/or managing children with tuberculosis, lay-workers performing symptom screening in facilities, engaging private sector primary-level providers and making treatment services available in a more decentralized type of health facility. Community-based approaches included home visits for contact screening and community-based treatment support. Some interventions included both facility-based and community-based activities, while others included only one or the other. In addition, nine interventions included community awareness campaigns or health system strengthening through provision of supplies or procurement support to health facilities; these activities did not clearly fall into one of the care models of interest but could have contributed to improved outcomes. Most interventions received support from international funders and introduced dedicated personnel or resources into the health system.

**Table 3:** Evidence for impact of decentralized care on child and adolescent tuberculosis diagnosis, treatment and prevention outcomes

Type of outcome, care delivery approach	Study	Intervention condition outcomes	Control condition outcomes	Effect estimate (95% CI)
**Diagnosis**				
Strengthen services in facilities and provide services or outreach in communities	EGPAF, 2018^46^	5865 cases in 20.6 months	2295 cases in 12 months	IRR: 1.49 (1.42–1.56)
	Islam et al., 2017^19^	231 cases in 24 months	65 cases in 12 months	IRR: 1.78 (1.35–2.34)
	Joshi et al., 2015^20^	360 cases in 12 months	113 cases in 12 months	IRR: 1.14 (0.83–1.56)^a^
	Khan et al., 2012^21^	205 cases in 12 months	28 cases in 12 months	IRR: 7.32 (4.39–10.87)
	Maha et al., 2019^22^	295 cases in 24 months	140 cases in 24 months	IRR: 2.11 (1.72–2.58)
	Malik et al., 2018^23^	1391 cases in 36 months	417 cases in 18 months	IRR: 2.96 (2.49–3.50)^a^
	Oshi et al., 2016^24^	1590 cases in 12 months	1210 cases in 12 months	IRR: 1.31 (1.22–1.42)
	Talukder et al., 2012^25^	175 cases in 24 months	130 cases in 24 months	IRR: 1.87 (1.28–2.71)
	Zawedde-Muyanja et al., 2018^26^	657 cases in 12 months	271 cases in 12 months	IRR: 2.39 (2.07–2.75)
Services in communities	Hanrahan et al., 2019^27^	189 cases in 18 months	216 cases in 18 months	IRR: 0.88 (0.31–2.46)
	Bayona et al., 2003^28^	1/151 (1%) contacts diagnosed with tuberculosis	3/118 (3%) contacts diagnosed with tuberculosis	RR: 0.26 (0.01–2.56)
	Fatima et al., 2016^29^	13 288 cases in 24 months	12 506 cases in 24 months	IRR: 1.06 (1.03–1.08)
	Davis et al., 2019^30^	8/216 (4%) contacts diagnosed with tuberculosis	10/227 (4%) contacts diagnosed with tuberculosis	RR: 0.84 (0.34–2.09)
	Moyo et al., 2012^31^	89/2381 (4%) children diagnosed with tuberculosis	36/2382 (2%) children diagnosed with tuberculosis	Ratio of event rate per person-year: 2.6 (1.8–4.0)^b^
	Reddy et al., 2015^32^	7 cases in 6 months	2 cases in 6 months	IRR: 0.71 (0.04–12.07)^a^
Strengthen services in facilities	Sachdeva et al., 2015^33^	271/2570 (11%) evaluated individuals diagnosed with tuberculosis	46/428 (11%) evaluated individuals diagnosed with tuberculosis	RR: 0.98 (0.72–1.33)
**Treatment**				
Strengthen services in facilities and provide services in communities	Zawedde-Muyanja et al., 2018^26^	310/382 (81%) patients with treatment success	121/186 (65%) patients with treatment success	RR: 1.25 (1.11–1.40)
Services in communities	Jeena & Naidoo, 2016^34^	65/82 (79%) patients with treatment success	52/97 (54%) patients with treatment success	RR: 1.48 (1.19–1.84)
	Mathew et al., 2005^35^	16/16 (100%) patients with treatment success	31/45 (69%) patients with treatment success	RR: 1.45 (1.19–1.77)
	Tripathy et al., 2013^36^	7/7 (100%) patients with treatment success	22/23 (96%) patients with treatment success	RR: 1.05 (0.96–1.14)
Strengthen services in facilities	Datiko & Lindtjørn, 2009^37^	21/23 (91%) patients with treatment success	8/9 (89%) patients with treatment success	RR: 1.03 (0.79–1.34)
**Prevention**				
Strengthen services in facilities and provide services in communities	EGPAF, 2018^46^	12 634 preventive treatment initiations in 20.6 months	1758 preventive treatment initiations in 12 months	8-fold increase in median monthly preventive treatment initiations per site, *P* < 0.001^b^
	Yassin et al., 2013^38^	698 preventive treatment initiations in 6 months	0 preventive treatment initiations in 15 months	Undefined
Services in communities	Zachariah et al., 2003^39^	25/113 (22%) identified contacts initiated preventive treatment	22/126 (17%) identified contacts initiated preventive treatment	RR: 1.27 (0.76–2.12)
Strengthen services in facilities	Oxlade et al., 2021^44^	12 preventive treatment initiations in 6 months	3 preventive treatment initiations in 6 months	IRR: 4.00 (1.08–22.09)

CI: confidence interval; EGPAF: Elizabeth Glaser Pediatric AIDS Foundation; IRR: incidence rate ratio; RR: risk ratio.

Note: We defined children and adolescents as people 0–19 years old, but individual studies focused on different age groups within this range.

^a^ Rate ratio adjusted for change in case notifications in a control area during the same time period.

^b^ This effect estimate reported by the study was more rigorous than what we could derive from available count data.

HIV: human immunodeficiency virus.

Note: Based on the 47 included studies.

Grouping studies by outcome and whether they contained facility-based service strengthening, community-based services or both yielded nine groups of studies of decentralized care (Table 3). The largest group comprised nine studies that simultaneously used facility-based interventions to improve the quality of diagnostic services in primary-level settings and community-based interventions to increase the likelihood that children or adolescents with tuberculosis would enter the health system.^19^^–^^26^^,^^46^ This combined facility and community approach consistently increased tuberculosis diagnoses in the 0–14 years age group, with effect sizes ranging from a one-to-sevenfold increase in diagnoses. In contrast, tuberculosis diagnoses did not generally increase in studies of interventions that screened people in their homes but referred them to existing health services for evaluation.^27^^–^^32^ We also observed this contrast among three tuberculosis preventive treatment studies. Large increases in preventive treatment initiation were achieved in the studies that simultaneously strengthened preventive treatment services in health facilities and provided home-based screening for contacts,^38^^,^^46^ but not in the study that used home-based screening alone.^39^ Improved treatment outcomes in the 0–14 year age group were observed for studies that included community-based treatment support, with at least 79% of patients in the intervention groups achieving treatment success.^26^^,^^34^^–^^36^ Five studies disaggregated results for young children versus older children and adolescents, but there was no clear consensus about whether interventions benefited these groups differentially based on the data presented (results available in the data repository).^16^^,^^20^^,^^26^^,^^27^^,^^30^^,^^34^

#### Integrated care interventions

We identified three comparative studies where integration was the primary intervention (Table 4).^40^^,^^41^^,^^45^ A stepped-wedge trial in Ethiopia showed that screening in integrated maternal, neonatal and child illnesses clinics increased tuberculosis diagnoses among children (0.5; 95% CI: 0.2–0.7; additional cases per clinic per 4-month period).^41^ A pre-post study from Zambia showed that having antiretroviral services and tuberculosis services in the same health facilities led to increased case notifications in the 0–14 year age group (IRR: 2.67; 95% CI: 1.05–6.76).^40^ A pre-post study from Kenya showed that screening in the maternal–child health, nutrition and acute care departments of a hospital did not significantly increase tuberculosis treatment registrations in the 0–14 year age group (IRR: 0.88; 95% CI: 0.44–1.77).^45^

**Table 4:** Evidence for impact of integrated care on child and adolescent tuberculosis diagnosis and treatment outcomes

Type of outcome, care delivery approach	Study	Intervention condition outcomes	Control condition outcomes	Effect estimate (95% CI)
**Diagnosis**				
Introducing ART services into health centres with tuberculosis services	Miyano et al., 2013^40^	40 tuberculosis patients registered during 3 intervention years (13 per year)	5 tuberculosis patients registered in pre-intervention year	IRR: 2.67 (1.05–6.76)
Tuberculosis screening in clinic or centre providing care for another condition	Ketema et al., 2020^41^	38/95 618 (4 per 10 000) children attending Integrated maternal, neonatal and child illnesses clinics were diagnosed with tuberculosis	9/85 278 (1 per 10 000) children attending integrated maternal, neonatal and child illnesses clinics were diagnosed with tuberculosis	0.5: (0.2–0.7) additional cases per clinic per 4-month period^a^
	Szkwarko et al., 2021^45^	15 tuberculosis patients registered after introducing screening programme in maternal and child health nutrition and acute care clinics	17 tuberculosis patients registered in pre-intervention period	IRR: 0.88 (0.44–1.77)
	Joshi et al., 2015^20^	0/700 (0%) children attending maternal–child health clinics diagnosed with tuberculosis	NR	NR
	Oshi et al., 2016^24^	133/2686 (5%) HIV clinic attendees diagnosed with tuberculosis	NR	NR
	Mehra et al., 2016^47^	0/6 (0%) HIV clinic attendees diagnosed with tuberculosis	NA	NA
	Qader et al., 2019^48^	4/467 (1%) mental health centre clients diagnosed with tuberculosis	NA	NA
	Szkwarko et al., 2016^49^	0/53 (0%) street-connected young people diagnosed with tuberculosis	NA	NA
**Treatment**				
Tuberculosis treatment and ART provided at the same clinic	Mandalakas et al., 2020^50^	857/1160 (74%) patients with treatment success	NA	NA
	Patel et al., 2013^51^	27/31 (87%) patients with treatment success	NA	NA
	Reif et al., 2018^52^	277/345 (80%) patients with treatment success	NA	NA

ART: antiretroviral therapy; CI: confidence interval; HIV: human immunodeficiency virus; IRR: incidence rate ratio; NA: not applicable; NR: not reported.

^a^ This effect estimate reported by the study was more rigorous than what we could derive from available count data.

Note: We defined children and adolescents as people 0–19 years old, but individual studies focused on different age groups within this range.

Among both comparative and non-comparative studies, integrated approaches to care included tuberculosis screening in clinics or centres for other health conditions and co-location of tuberculosis and HIV treatment (Fig. 2). Tuberculosis screening was performed in HIV clinics, maternal–child health clinics, nutrition clinics, mental health centres and a centre providing services to street-connected young people. In general, the proportion of screened children diagnosed with tuberculosis in integrated interventions was small, except for screening conducted in an HIV clinic.^24^ Three non-comparative studies described delivering both tuberculosis treatment and antiretroviral therapy in a single clinic to children and adolescents living with HIV;^50^^–^^52^ the proportion with successful treatment in these studies ranged from 74% to 87%.

#### Family-centred care interventions

We identified two comparative studies where family-centred care was the primary intervention (Table 5). A cluster-randomized trial from Peru showed that providing socioeconomic support to families affected by tuberculosis increased the proportion of contacts aged 0–19 years who initiated preventive treatment (RR: 1.70; 95% CI: 1.10–2.64).^42^ An earlier pre-post study from the same setting showed that socioeconomic support improved both preventive treatment initiation (RR: 2.23; 95% CI: 2.11–2.36) and preventive treatment completion (RR: 3.22; 95% CI: 2.90–3.57).^43^ Among both comparative and non-comparative studies, family-centred approaches fell into two main categories: support for patients and families; and patient choice (Fig. 2). Support strategies included transport enablers to help people get to health facilities, food or nutritional supplements, cash transfers, support for income-generating activities, psychological counselling and establishing peer groups of people affected by tuberculosis to promote mutual support and empowerment. Patient choice strategies included considering patient preferences for treatment supporter, location and regimen. Five studies that provided socioeconomic or psychosocial support during treatment reported treatment success among at least 71% of children and adolescents with tuberculosis.^55^^–^^58^^,^^62^

**Table 5:** Evidence for impact of family-centred care on child and adolescent tuberculosis diagnosis, treatment and prevention outcomes

Type of outcome, care delivery approach	Author	Intervention condition outcomes	Control condition outcomes	Effect estimate (95% Cl)
**Diagnosis**				
Support	Htet et al., 2018^53^	10/59 (17%) contacts diagnosed with tuberculosis	NA	NA
	Imsanguan., et al., 2020^54^	3/48 (6%) contacts diagnosed with tuberculosis	NA	NA
Choice	Kay et al., 2022^65^	3/492 (1%) contacts diagnosed with tuberculosis	NA	NA
**Treatment**				
Support	Demissie et al., 2003^55^	5/7 (71%) patients with treatment success	NA	NA
	Oyieng’o et al., 2012^56^	1/1 (100%) MDR-tuberculosis patient with treatment success	NA	NA
	Satti et al., 2012^57^	15/17 (88%) MDR-tuberculosis patients with treatment success	NA	NA
	Wai et al., 2017^58^	1/1 (100%) MDR-tuberculosis patient with treatment success	NA	NA
Choice	Dick et al., 1996^59^	148/203 (73%) patients classified as adherent	NA	NA
	Soomro et al., 2012^60^	14/14 (100%) patients with treatment success	NA	NA
	van den Boogaard et al., 2009^61^	245/308 (80%) patients with treatment success	NA	NA
Support and choice	Mesic et al., 2020^62^	8/11 (73%) patients with rifampicin-resistant tuberculosis with treatment success	NA	NA
**Prevention**				
Support	Wingfield et al., 2017^42^	91/206 (44%) identified contacts initiated preventive treatment	53/206 (26%) identified contacts initiated preventive treatment	RR: 1.70 (1.10–2.64)
	Rocha et al., 2011^43^	477/542 (88%) identified contacts initiated preventive treatment	1116/2929 (39%) identified contacts initiated preventive treatment	RR: 2.23 (2.11–2.36)
		383/441 (87%) contacts completed preventive treatment	301/1116 (27%) contacts completed preventive treatment	RR: 3.22 (2.90–3.57)
	Yuen et al., 2019^63^	57/140 (41%) identified contacts initiated preventive treatment	NA	NA
		51/57 (89%) contacts completed preventive treatment	NA	NA
Choice	Triasih et al., 2016^64^	86/99 (87%) eligible contacts initiated preventive treatment	NA	NA
		50/86 (58%) contacts completed preventive treatment	NA	NA
	Kay et al., 2022^65^	237/248 (96%) contacts completed preventive treatment	NA	NA

CI: confidence interval; MDR: multidrug resistant; NA: not applicable; RR: risk ratio.

Note: We defined children and adolescents as people 0–19 years old, but individual studies focused on different age groups within this range.

### Evidence gaps

We found limited reports assessing the impact of integrated and family-centred care models on children and adolescents affected by tuberculosis, although non-comparative studies suggest that programmes are providing integrated and family-centred care to children and adolescents. Many studies that may have included children and adolescents did not sufficiently age-disaggregate data to allow assessment of child and adolescent outcomes. Even where data were age-disaggregated, the conventional use of 0–14 years as the youngest age group meant that we were unable to separate child from adolescent outcomes, and outcomes for older adolescents were generally aggregated with an adult age group.

## Discussion

Our review identified a large variety of decentralized, integrated and family-centred care approaches for children and adolescents with tuberculosis disease and exposure. We found substantial evidence that simultaneously strengthening diagnostic services in decentralized health facilities and strengthening the linkages between communities and these facilities improves case detection, but doing only one or the other does not. More limited evidence suggests that strengthening decentralized preventive treatment services and providing socioeconomic support to families improves preventive treatment initiation and completion. Consistent with a previous review,^5^ we found that community-based treatment support improves tuberculosis disease treatment outcomes. Finally, integrated and family-centred approaches are being used by programmes in diverse settings and are achieving good outcomes, despite a dearth of formal impact evaluations of these approaches.

Our findings highlight a couple of key considerations for the design of care models that improve child and adolescent tuberculosis outcomes. One consideration is the importance of reducing barriers simultaneously in the community and in facilities. Doing only one or the other may in some instances improve tuberculosis case detection among adults but not children, given that adults can more readily be diagnosed by sputum testing.^29^^,^^33^ However, reducing barriers in communities and facilities for the benefit of children and adolescents may benefit adults as well.^66^ Another consideration is the importance of introducing new resources or workers to improve child and adolescent tuberculosis care. Funding is required to pay dedicated staff, provide transport enablers for community health workers or patients, provide supplies or equipment to health facilities or provide material support to patients and their families. When funding for these services becomes unavailable, programmatic gains may be lost.^67^ If health system strengthening is incorporated into an intervention strategy, then positive impact may be maintained even after a time-limited activity (e.g. a mass community screening effort) ends.^68^

Our review also identified two key priority areas for future research. One area is identifying effective integrated or family-centred care approaches for children and adolescents. Ongoing studies are evaluating the integration of tuberculosis services with child health care services^69^ and shared decision-making for preventive treatment delivery location.^70^ Another potential approach that could be evaluated is providing coordinated care to an entire family through shared medical appointments or counselling sessions, which could be particularly useful in the context of expanding preventive treatment to contacts of all ages. A second area is care models for adolescents. Adolescents have unique health needs that require youth-centred strategies, particularly for diseases requiring prolonged or long-term treatment.^71^ Adolescent-centred strategies are being increasingly studied in the HIV field.^72^^,^^73^ However, tuberculosis services have generally not yet been tailored to adolescent needs,^74^^,^^75^ and our review identified only two studies that provided adolescent-centred services.^49^^,^^52^

A major limitation of our review is that the lack of clear definitions for the care models of interest made development of search terms challenging. However, strengths of our approach were the iterative process used to identify relevant literature, developed in consultation with people who work in tuberculosis programmes in countries with high tuberculosis burdens and the extensive use of existing systematic reviews. Even so, it is possible that we missed studies that described the care models of interest in ways that we did not consider. Another limitation is that we did not contact authors following article identification to request age-disaggregated data given time constraints, as we had a firm deadline aligned to the inputs into the WHO consolidated guidelines on the management of tuberculosis in children and adolescents.^76^ Authors of a previous systematic review of adherence support interventions for tuberculosis disease treatment in children did contact authors and consequently were able to compile a more comprehensive evidence base for this specific set of interventions.^5^ Finally, we limited our review to studies from countries with high prevalence of tuberculosis in an effort to identify the most relevant interventions for these settings. However, in doing so, we likely missed studies from countries that might have had more resources to conduct research around care delivery and where tuberculosis preventive treatment for adolescents is more common.

Our findings suggest that local and international policy-makers and funders should invest in decentralized, integrated and family-centred tuberculosis services to close the large case detection and prevention gaps for children and adolescents. Policies for decentralization should articulate strategies to strengthen services in both primary-level health facilities and communities, thereby promoting linkages to tuberculosis care. Module 5 in *WHO operational handbook on tuberculosis* provides practical guidance for programmes seeking to implement the approaches summarized in this review.^77^ Future research should focus on identifying which approaches to integrated and family-centred care for children and adolescents are effective in different settings and health systems and evaluating the impact of these approaches. When programmes identify successful approaches, decision-makers must provide resources to ensure large-scale uptake and sustainability.
